# Tracking Electrons in Biological Macromolecules: From Ensemble to Single Molecule

**DOI:** 10.3390/molecules190811660

**Published:** 2014-08-06

**Authors:** Leandro C. Tabares, Ankur Gupta, Thijs J. Aartsma, Gerard W. Canters

**Affiliations:** 1 Commissariat à l’Energie Atomique, Institut de Biologie et de Technologies de Saclay, Service de Bioénergétique, Biologie Structurale et Mécanismes (CNRS UMR-8221), Gif-sur-Yvette Cedex 91191, France; 2 Leiden Institute of Physics, Huygens-Kamerlingh Onnes Laboratory, Leiden University, Niels Bohrweg 2, PO Box 9504, RA Leiden 2300, The Netherlands

**Keywords:** electron transfer, random sequential, single molecule, enzyme, FRET, redox, nitrite reductase, small laccase, copper protein, ABEL trap

## Abstract

Nature utilizes oxido-reductases to cater to the energy demands of most biochemical processes in respiratory species. Oxido-reductases are capable of meeting this challenge by utilizing redox active sites, often containing transition metal ions, which facilitate movement and relocation of electrons/protons to create a potential gradient that is used to energize redox reactions. There has been a consistent struggle by researchers to estimate the electron transfer rate constants in physiologically relevant processes. This review provides a brief background on the measurements of electron transfer rates in biological molecules, in particular Cu-containing enzymes, and highlights the recent advances in monitoring these electron transfer events at the single molecule level or better to say, at the individual event level.

## 1. Introduction

The application of fluorescence-based single molecule (SM) techniques to the study of enzymes opened a new field of research from the 1980s onwards [1,2]. Although in 1961 Rotman already successfully performed a series of elegant experiments on single molecules of glucose oxidase [3], the combination of laser excitation and confocal microscopy by Rigler in 1991 represented a major breakthrough and put single molecule research on a firm footing [4,5,6]. Enzymes were often studied by fluorescent labeling, whereas sometimes the intrinsic fluorescence of the protein could be used as such. In the latter case the fluorescence as a rule originates from aromatic amino acids and fluorescence excitation must rely perforce on the use of (near)-UV excitation, where laser sources are scarce. Single molecule studies of enzymes and proteins therefore have relied prevalently not on intrinsic fluorescence, but on fluorescent labeling or on the use of fluorescent or fluorogenic substrates.

**Figure 1:**
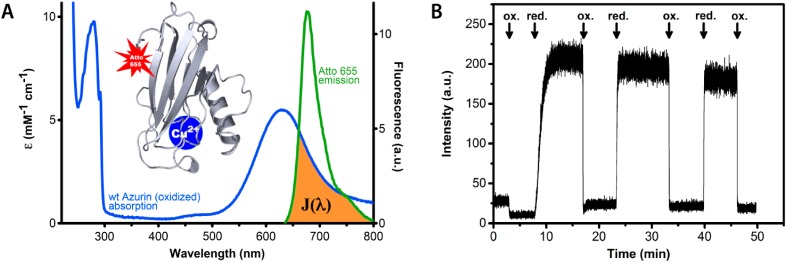
Redox state dependent quenching of the fluorescence of a dye label covalently attached to the blue copper protein azurin from *Pseudomonas aeruginosa*. (**A**) Absorption and emission spectra of azurin and label, respectively. The blue curve represents the absorption spectrum of oxidized azurin, the green curve is the fluorescence spectrum of the Atto-655 dye. The brown colored area symbolizes the spectral overlap between the dye emission and the protein absorption. The absorption band around 600 nm is absent when the redox center of azurin is reduced from Cu^2+^ to Cu^+^. The inset shows a protein backbone ribbon presentation of the azurin structure with a site-specifically attached fluorescent label (Atto-655). (**B**) The fluorescence intensity of Atto-655-labeled azurin in solution (25 nM) is high in the reduced state and low in the oxidized state. The arrows mark the addition of excess oxidant (hexacyano-ferrate(III)) and reductant (ascorbate). In the oxidized state the fluorescence is quenched by energy transfer from the Atto-655 label to the Cu-center.

We and others have shown that fluorescent labeling of oxido-reductases makes these proteins eminently suitable for SM studies by means of Förster resonant energy transfer (FRET)-related quenching of the fluorophore [7,8,9]. This is possible because the active center(s) of these proteins often have strong characteristic features in their optical absorption spectra which are redox state dependent. The fluorescence intensity of a properly chosen and covalently attached dye label can therefore be quenched by FRET between label and redox center to an extent that depends on the redox state of the protein. A simple example is provided by the small blue copper protein azurin (Figure 1) labeled by the dye Atto-655. *In vivo* this protein functions as an electron shuttle by means of its copper containing active site, that may occur in the reduced (Cu^+^) and the oxidized (Cu^2+^) form. In the latter case the protein exhibits a strong absorption band around 600 nm, hence its blue color. As shown in Figure 1, the dye emission is strongly dependent on the redox state of the protein. When the protein is in the oxidized state, FRET between the dye and the Cu center largely quenches the dye fluorescence whereas quenching is absent when the protein is in the reduced state and its 600 nm absorption has vanished.

**Figure 2:**
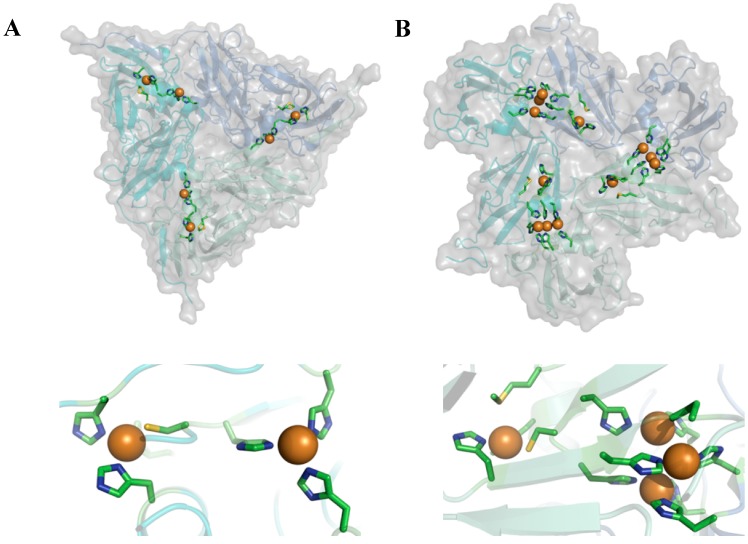
Cartoon representation depicting the overall structure (top) and active site (bottom) of nitrite reductase from *Alcaligenes xylosoxidans* (**A**) (pdb: 1oe1) and small laccase from *Streptomyces coelicolor* (**B**) (pdb: 3cg8).

We have used dye labeling to study the catalytic mechanisms of two copper containing enzymes, the nitrite reductases (NiRs) from *Alcaligenes faecalis-*S6 and *Alcaligenes xylosoxidans*, and the small laccase (SLAC) from *Streptomyces coelicolor*. These two types of enzyme are interesting because they have similar homo-trimeric structures (Figure 2) but different functionalities. They catalyze the conversion of nitrite (NO_2_^−^) to NO and of O_2_ to water, respectively. In both cases reducing equivalents are taken up by a so-called T1 Cu center and transferred to the catalytically active site which consists of a single so-called T2 Cu center in NiR and a trinuclear center (TNC) in the case of SLAC. In both enzymes the latter centers are located between two monomers in the 3D-structure of the enzyme (Figure 2).

In the case of NiR one of the central research issues concerns the question whether the enzyme functions according to an “ordered mechanism” or according to a “random sequential mechanism” (*vide infra*). In the case of the multi-copper oxidases the mechanism of electron transfer and of O_2_ reduction has been the subject of a long standing debate [10,11,12,13]. Our research on SLAC has focused on the electron transfer between the T1 center and the TNC during turn-over of the enzyme. Here we briefly review the results of SM investigations on NiR and SLAC over the past decade.

## 2. Nitrite Reductase

As mentioned above, NiR catalyzes the conversion of NO_2_^−^ to NO. A type 1 (T1) copper site acquires reducing equivalents (electrons) from a reducing substrate, which are then transferred to a type 2 (T2) copper site where NO_2_^−^ is reduced (NO_2_^−^ + e^−^ + 2H^+^ → NO + H_2_O) [14]. The dispute about the enzyme mechanism revolves around the question whether NO_2_^−^ binds to the T2 copper site only after the latter has received an electron from the T1 copper site (“reduction first” mechanism) [14,15,16,17,18] or whether the electron is transferred only after binding of NO_2_^−^ to the T2 copper site (“binding first” mechanism) [18,19,20,21,22,23,24]. Both cases are referred to as “ordered mechanisms”. Conversely it has been argued by Wijma *et al*., that the enzyme operates according to a random sequential mechanism by which either pathway is feasible, with the preponderant route depending on parameters such as pH and nitrite concentration [25,26,27].

**Figure 3:**
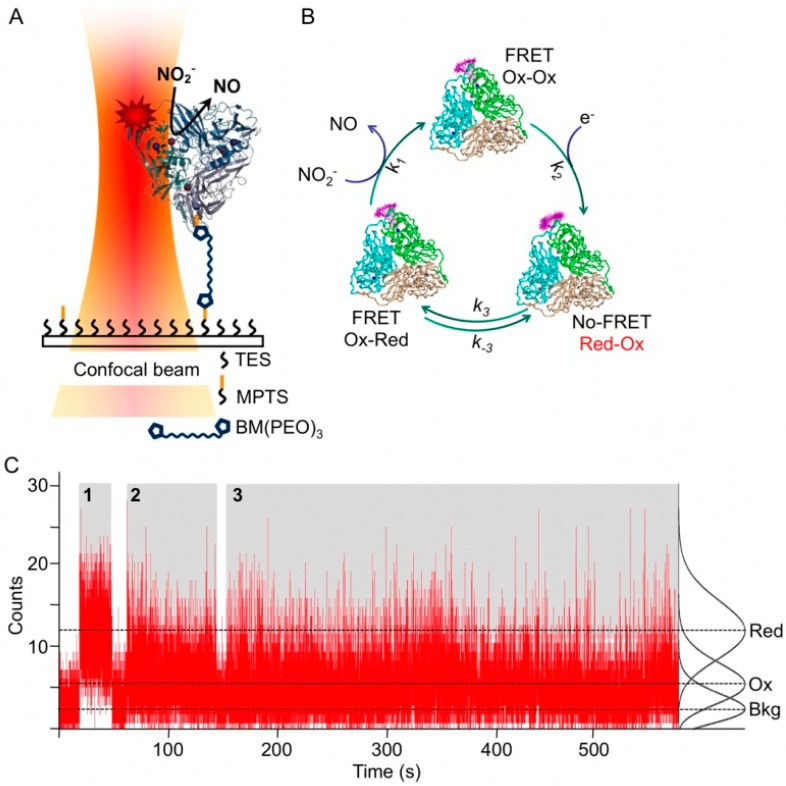
Single molecule experiments on Af-NiR-A655. (**A**) Enzyme immobilization scheme. Af-NiR-A655 is immobilized through a 1,11-bis-maleimidotriethyleneglycol (BM(PEO)_3_) linker to the glass cover slip modified with a 100:1 mixture of triethoxy (TES) and mercaptopropyl trimethoxysilane (MPTS). The BM(PEO)_3_ linker was attached to the protein via an exposed cysteine, introduced at position 93. The glass cover slip is covered with buffer to which nitrite plus a mixture of ascorbate/phenazine ethosulfate (PES) as sacrificial electron donor were added [28]; (**B**) Scheme of events during the turnover of labeled NiR. In the resting enzyme both T1 Cu and T2 Cu are oxidized (Ox-Ox) and dye fluorescence is partially quenched. Upon reduction of T1 Cu (Red-Ox) the dye fluorescence goes up as FRET to the reduced T1 Cu is not possible. When an electron is transferred from T1 to T2 Cu the label fluorescence is quenched again by the oxidized T1 Cu (Ox-Red). Nitrite binds to the reduced T2 Cu, is converted into nitric oxide and dissociates from the enzyme that goes back to the resting form; (**C**) Fluorescence time traces of individual Af-NiR-A655 molecules. The number of photons emitted during 10 ms intervals is recorded as function of time for three independent molecules during time blocks denoted by 1, 2 and 3 and colored in grey. The recording starts at the left of each time block and ends at the right with the bleaching of the molecule. Recording 1 represents a fully reduced inactive molecule while 2 and 3 represent turning over enzymes. The black curves at the right are the normalized Poissonian fits of the intensity distribution histograms and correspond with background (Bkg), oxidized (Ox) and reduced (Red) Af-NiR-A655. The dotted lines visualize the corresponding three different fluorescence levels.

With the aim of shedding new light on this controversy and to gain new insight in the reaction mechanism of NiR it was decided to study this enzyme at the single molecule level. To this end NiR was labeled with a fluorescent dye whose emission spectrum overlaps with the absorption spectrum of the T1 Cu of NiR in the oxidized form. As the reduced form has no absorption features in the visible region, fluorescence is (partly) quenched through FRET only in the oxidized form. As a result, the fluorescence of a single molecule fluctuates during turn-over reflecting the fluctuations in the redox state of the T1 Cu (Figure 3C). In a first attempt NiR from *A. faecalis* was singly labeled with the dye Atto-655 (Af-NiR-655) and immobilized through a linker onto a modified glass surface (Figure 3A) [28].

Two types of fluorescence time traces were observed [28]. There were traces for which the fluorescence intensity was constant, *i.e.*, the intensity histogram fitted to a single Poisson distribution; they were attributed to inactive (T1 Cu reduced) molecules (Figure 3C). There were also traces for which two Poisson distributions were needed to fit the intensity histograms, indicating that the molecules were switching between two fluorescence levels (Figure 3C). These traces were attributed to catalytically active Af-NiR, with the lower level corresponding to the state in which the T1 Cu was oxidized and the higher level to the state in which the T1 Cu was reduced. The probability P_Red_ of finding the T1 Cu in the reduced form could then be calculated from the integral of the Poisson distribution. It was observed that P_Red_ decreased with increasing nitrite concentration. This is consistent with the expectation that the T1 Cu on average will stay longer in the reduced form at low NO_2_^−^ concentrations.

**Scheme 1:**
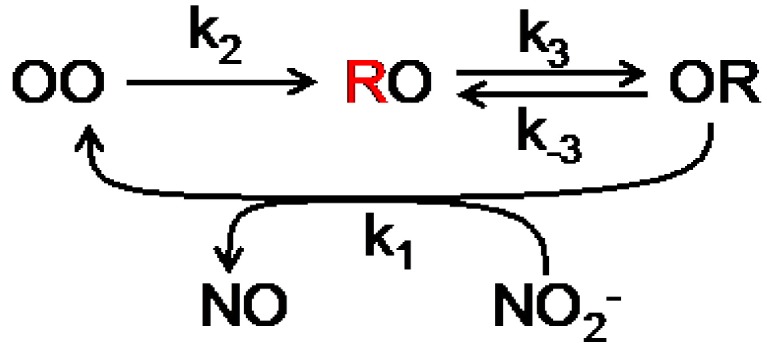
Scheme the catalytic cycle of NiR. The possible combinations of redox states of the T1 and T2 Cu centers are represented by OO (both oxidized), RO (T1 reduced, T2 oxidized) and OR (T1 oxidized, T2 reduced). The unquenched state is in red (T1 reduced). The external electron donor has been omitted from the first reduction step.

An autocorrelation analysis was used to extract kinetic information. The enzyme cycle of NiR can be represented by a three state cyclic reaction scheme (Scheme 1; see also Figure 3B). For such a scheme the first order autocorrelation function follows a single exponential decay [28,29]. Nevertheless, a stretched exponential function was needed to properly fit the experimental data indicating that NiR does not operate according to a single rate but rather exhibits a distribution of rates. This distribution narrowed with increasing nitrite concentration and exhibited saturation towards high concentrations of NO_2_^−^. From the fit the averaged kinetic constants of the Af-NiR catalytic cycle could be obtained: *k*_1_ = (4 ± 2) × 10^5^ M^−1^ s^−1^, *k*_2_ = 10 ± 3 s^−1^, *k*_3_ = 21 ± 6 s^−1^, *k*_−3_ = 14 ± 4 s^−1^. This work resolved for the first time the intramolecular electron-transfer (ET) rates *k*_3_ and *k*_-3_ during steady state turn-over. Previous values, obtained in pulse radiolysis studies, showed higher ET rates, in the order of 450 to 2100 s^−1^ (*k*_3_ + *k*_−3_) [30]. However, these were obtained in the absence of nitrite and cannot be compared directly with the rates obtained in the single molecule experiments. From the single molecule rates Michaelis-Menten parameters of *K*_M_ = 31 ± 17 µM and *V*_max_ = 6.5 ± 0.2 s^−1^ were obtained, in good agreement with the values measured in bulk [31].

**Figure 4:**
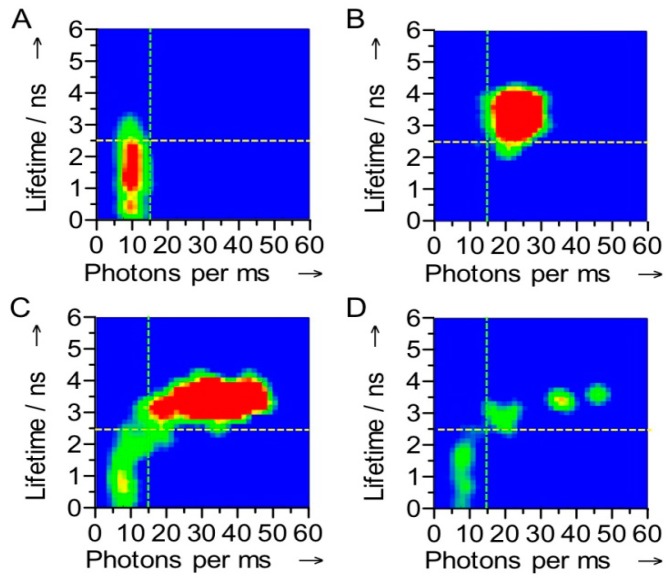
Correlograms of the fluorescence intensity and the fluorescence lifetime of a single Az-NiR-647N molecule. The measurements were performed under oxidizing (**A**), reducing (**B**) and turnover conditions (**C**,**D**). The stippled lines show how the molecules were divided into ox and red in the FLIM experiments (see text). The data were obtained with 4 ms binning times. The color is indicative of the frequency of occurrence in the histograms, a change from green to red corresponding with an increase in occurrence.

For Atto-655 labeled Af-NiR the fluorescence switching ratio (SR ≡ [*F*_red_ − *F*_ox_]/*F*_red_, *F*_red_ and *F*_ox_ being the fluorescence intensities with the enzyme T1 site in the red or the ox state, respectively) amounted to 50%. A higher contrast between the red and ox states would provide better opportunities to investigate the working of the enzyme. A follow-up study was performed on NiR from *Alcaligenes xylosoxidans*. In this “blue” NiR the T1 copper site exhibits a more pronounced absorbance around 600 nm when oxidized. When labelling this enzyme with Atto-647N (Ax-NiR-A647N) a switching ratio of almost 90% was observed [32]. The switching was also evident in the fluorescence lifetime: with Ax-NiR-A647N in the reduced state the dye fluorescence lifetime amounted to 3.7 ns, comparable to that of the free dye, whereas in the oxidized form it amounted to only 1.1 ns. At the single molecule level a correlation between the number of photons emitted and the fluorescence lifetime was observed (Figure 4). Although in oxidizing or reducing conditions a narrow distribution for each state was observed, under turnover conditions the correlograms showed that the molecules fluctuate between the oxidized and reduced form (Figure 4).

**Figure 5:**
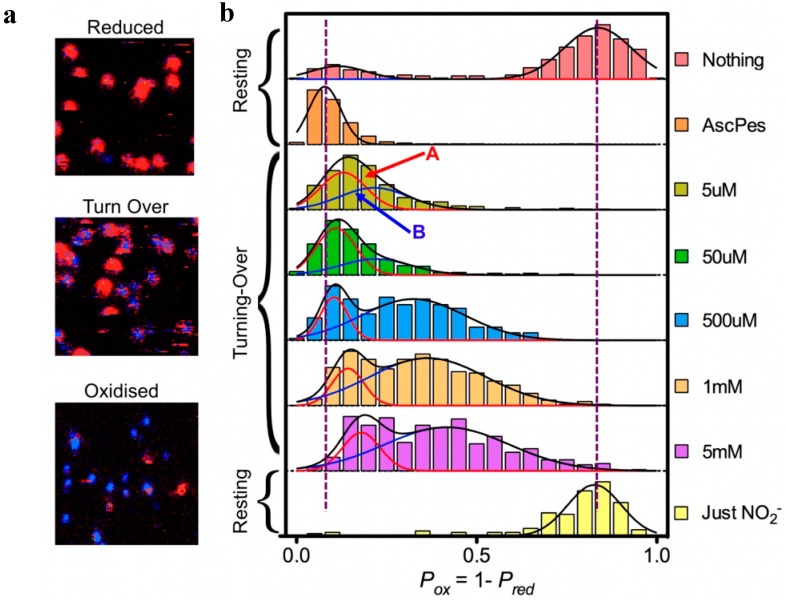
FLIM experiments on Ax-Nir-647N. (**a**): Part (7 × 7 μm) of surface area scanned row by row from right to left and from top to bottom; pixel size 75 nm, dwell time per pixel 4 ms. Ax-NiR-647N was immobilized in an agarose gel on the surface of a glass cover slip [32], in the presence of 10 mm ascorbate and 100 nm PES (“Reduced”), 10 mm ascorbate, 100 nm PES, and 500 mm NaNO_2_ (“Turn Over”) and 5 mm NaNO2 (“Oxidised”). After fitting, pixels were colored red (lifetimes > 2.5 ns) or blue (lifetimes < 2.5 ns). For each spot the probability of finding the NiR reduced (P_red_) was calculated as the number of red pixels weighted by the total number of pixels in the spot. (**b**): Histograms of P_ox_ for freshly prepared enzyme in pH 7.5 buffer with no reductant or oxidant present (“Nothing”); under reducing conditions (“AscPes”); under turnover conditions with amounts of NaNO_2_ varying from 5 µM to 5 mM; and under oxidizing conditions (“Just NO_2_^−^”). The histograms were each fit to a single Gaussian (“AscPES” and “Just NO_2_^−^”) or to a sum of two Gaussians for the other cases. Molecules corresponding to Population A (reduction first) and B (binding first) are indicated by red and blue arrows, respectively.

This correlation between fluorescence intensity and lifetime allowed the use of Fluorescence Lifetime Imaging Microscopy (FLIM). By scanning the glass surface containing the immobilized Ax-NiR-A647N molecules under a confocal microscope and recording the fluorescence life time pixel by pixel single turning-over enzyme molecules could be visualized in a fast raster-scanning mode (Figure 5). Surprisingly these pictures showed two populations of molecules. Molecules in one population (say “A”) stayed reduced most of the time while for the other one (population “B”) the molecules were switching between the reduced and the oxidized form at a rate comparable to that of the scanning (4 ms per pixel). The ratio between these two populations could be changed by changing the NO_2_^−^ concentration. The results were consistent with the previous proposition by Wijma *et al*., that NiR operates according to a random sequential mechanism [25], molecules in population A following the binding first route, whereas B molecules would follow the reduction first route [32].

**Figure 6:**
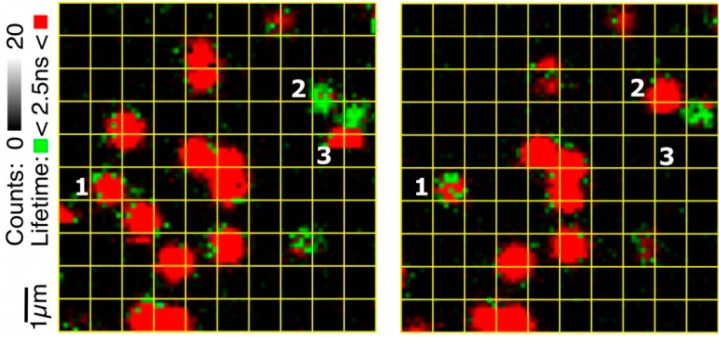
Example of switching between populations. The two images were taken of the same area of the glass surface carrying the Af-NiR-647N molecules immobilized in agarose with an interval of 10 min (left first). The grid (1 × 1 μm) has been applied to facilitate comparing the positions of the molecules in the two images. Molecule 1 switches from population A to B while the opposite happens for molecule 2. A few molecules disappear in the course of time due to bleaching. For instance, in the left image a single-step photo-bleaching occurs for molecule 3 during the scan. Experimental conditions: 10 mM ascorbate, 100 nM PES, 500 mM NaNO_2_; 100 mM potassium phosphate, pH 7.5.

The important contribution from the single molecule experiments is that they provided a better understanding of the meaning of “random”. Although from in-bulk experiments one might think that a given enzyme molecule may stochastically chose between the reduction first and the binding first path at each turn-over, the SM experiments demonstrate that “randomness” rather relates to a stochastic distribution of the molecules over the two populations. A molecule in population A would follow the binding first pathway for at least few minutes before switching to population B and start following the other pathway (Figure 6). This is a remarkable observation for the explanation of which at present no adequate mechanistic model is available

The FLIM experiments clearly showed that two populations were coexisting, each of them possibly corresponding to one of the proposed paths for the reduction of nitrite. However the experiments lacked the necessary temporal resolution to obtain kinetic parameters. Moreover, the molecules were immobilized in an agarose gel raising the question of whether the heterogeneity could be an artefact.

To address these points a novel approach was chosen in collaboration with Moerner and co-workers in Stanford (USA) [7]. Using a specialized microfluidic trapping device that cancels the Brownian motion of a single emissive solution-phase object through the use of directed electro-osmotic forces, a so-called anti-Brownian electrokinetic (ABEL) trap, [33] we were able to “trap” and observe for the first time the turn-over of an enzyme in solution at the single molecule level.

**Figure 7:**
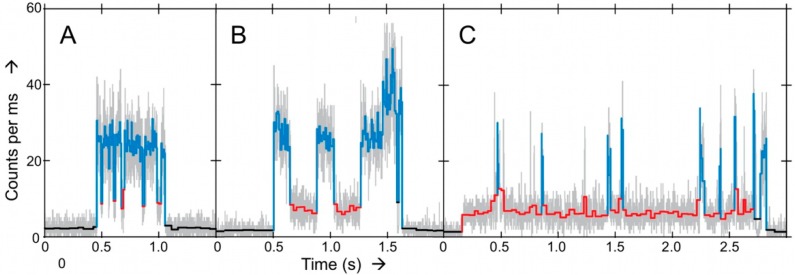
Panels **A**–**C** represent examples of intensity-time traces of three different Ax-NiR-A647N molecules trapped in solution with different turnover behavior [7]. Grey traces represent the 1 ms binned data. In color the results obtained from applying a change-point-finding algorithm as a filter on time-tagged data. Three levels were found: upper level corresponding to the reduced form (light blue), a lower level corresponding to the oxidized form (red) and the background (black). Conditions: ascorbate 5 mM; PES, 100 nM; and nitrite 5 mM. Copyright 2011 National Academy of Sciences USA.

In these experiments Ax-NiR-A647N was mixed in the microfluidic cell with ascorbate as a reductant plus phenazine-ethosulfate as a mediator (Asc/PES) and NO_2_^−^ as the substrate. When an Ax-NiR-A647N molecule was trapped it was possible to observe its turning-over during many cycles for several seconds. Different from the Af-NiR-A655 experiment (*vide supra*), the oxidized and reduced levels could be easily distinguished (Figure 7).

**Scheme 2:**
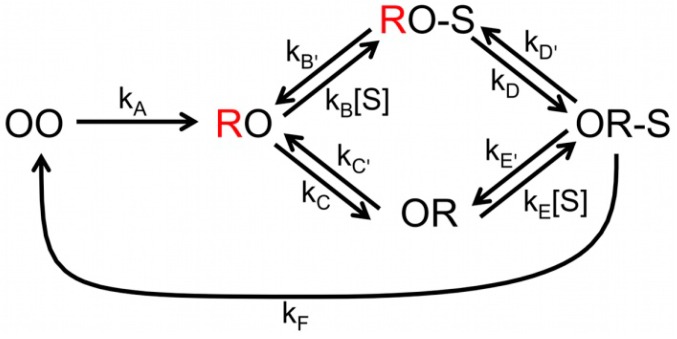
Complete scheme of NiR catalytic cycle. The possible combinations of redox states of the T1 and T2 Cu centers are represented by OO (both oxidized), RO (T1 reduced, T2 oxidized) and OR (T1 oxidized, T2 reduced). The unquenched states are in red (T1 reduced). NO_2_^−^ is represented by S; the external electron donor has been omitted.

As in the previous study, no single kinetic behavior was observed, pointing again to heterogeneity in the NiR population. This time the better time resolved data allowed a more sophisticated analysis. The dwell times in the reduced and oxidized states were analyzed by means of a bin-free state identification procedure. This comprised first the application of an intensity-change-point-finding algorithm as a smart low-pass filter directly on time-tagged data [34]. This was followed by applying an intensity thresholding algorithm to identify the states. Initially, by fitting the dwell time distributions to Scheme 1 the intramolecular ET rate (*k*_3_) could be extracted. Interestingly, *k*_3_ increased with the nitrite concentration. This is in agreement with a random-sequential mechanism whereby ET between the T1 and T2 sites may occur before or after binding of NO_2_^−^. The relative contribution of each path to the overall ET kinetics depends on environmental variables such as substrate concentration and pH. But it also means that Scheme 1 is too simplistic for an adequate analysis of the data. A more complex model had to be used that explicitly considers substrate binding and the two proposed pathways (Scheme 2).

By globally fitting all the data according to Scheme 2 estimates of all the kinetic parameters could be obtained [7] As already concluded from the FLIM experiments, the analysis of the ABEL data confirms that at low substrate concentration the majority of the molecules follows the “reduction first pathway”. However, as substrate concentration increases an increasing fraction of molecules follows the “binding first” pathway. It should be noted, that although the observations pertained to individual bNiR molecules, the global analysis provided only rate constants that are averaged over many molecules. Obtaining rates for individual molecules requires a substantially longer viewing window than could be achieved in the present experiments. This could be realized successfully in the experiments on SLAC (see next section). Improvement in the design of the ABEL trap may hold promise of extending the viewing window. This together with the study of the effect of changes in the reductant concentration and pH on the enzyme kinetics may allow us to gain a still deeper understanding of the reaction mechanism of NiR’s.

## 3. Small Laccase (SLAC)

SLAC belongs to the family of multicopper oxidases (MCOs) which contain four Cu ions in their active sites. In addition to the T1 and T2 Cu ions found at the active site of NiR, MCOs contain an additional binuclear type 3 (T3) Cu pair located close to the T2 Cu. Together the latter form a trinuclear Cu cluster (TNC) (see Figure 2). Unlike NiRs, MCOs accept four reducing equivalents to convert O_2_ to H_2_O. They are received at the T1 Cu, one at a time, and transferred to the TNC via a conserved HisCysHis pathway. When O_2_ binds at the TNC it will get reduced to water in one or two steps completing a single turnover.

Most of the MCO’s, *viz*. ascorbate oxidase, Fet3p and the laccases, are monomers built up out of three cupredoxin-like domains. The overall structure of SLAC is different but it is similar to that of NiR in that SLAC is a homotrimer (see Figure 2). Recently, it has been proposed that not only the structure but also the mode of operation of SLAC may differ from the other MCO’s in the sense that SLAC may utilize a redox active tyrosine residue, Y108, during O_2_ reduction, in particular under conditions where there is a shortage of reducing equivalents in the milieu [35,36]. On the basis of a phylogenetic analysis of multicopper blue proteins, including the NiRs and the MCOs, an evolutionary relationship has been proposed between the latter two enzymes [37], which makes one all the more curious as to the mechanistic (dis)similarities among these proteins.

Here, we review the recent progress in measuring the rate of intramolecular ET from T1 to TNC, and back, during enzyme turnover. The ET process has been studied previously for a number of MCOs and NiRs. Experiments under steady state turn-over conditions were often difficult to analyze and interpret. ET rates were also studied under pre-steady-state conditions by single shot experiments using techniques like pulse radiolysis [38,39,40,41], flash photolysis [42], *etc.*, in attempts to study the reduction half cycle of the MCOs but the results have sometimes been inconsistent with those from steady state turn-over measurements [38,40], thus creating the need of new methods to study the ET rates. SM techniques allow the study of the intramolecular ET process under steady state turnover in a fairly straightforward and simple manner. This single molecule based approach allows to monitor the enzyme dynamics of single SLAC molecules at the individual molecular level.

**Figure 8:**
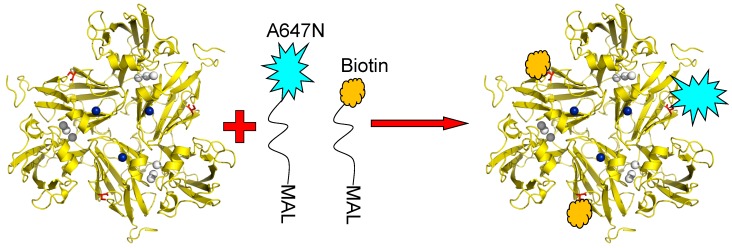
K204C variant of SLAC conjugated with Atto647N-maleimide and biotin-peg-maleimide. The conditions are chosen so that the labeling ratio of the dye to protein doesn’t exceed 5% to ensure most enzyme molecules carry only one or no fluorescent label. T1 Cu is depicted in blue, TNC in grey and Cys204 in red. Reprinted with permission from [43]. Copyright 2014 The Americal Chemical Society.

The principle utilized to monitor the redox state of the T1 Cu site by fluorescent labelling has already been introduced above. SLAC contains no cysteine residues other than C288 which is coordinated to the T1 Cu and, thus, there is no amino acid available in the structure for conjugation with a fluorophore or with other molecules like linkers. Therefore, variants of SLAC were prepared containing the K204C mutation so that a cysteine residue would be available at the protein surface for covalent linking to other molecules via thiol-maleimide chemistry. Instead of confining NiR molecules in an agarose matrix or an ABEL trap (see previous Section), we bound the SLAC molecules covalently in a preferred orientation to a transparent support. To this end the surface exposed cysteine residues of the SLAC K204C variant were conjugated to Atto647N and to biotin linkers. Since SLAC is a homotrimer three cysteines would be available for conjugation; thus, the reaction was carried out under conditions which would promote Atto647N conjugation to at most one cysteine and conjugation of a biotin-PEG linker to the remaining cysteines. A cartoon depicting the labelling strategy is shown in Figure 8.

**Figure 9:**
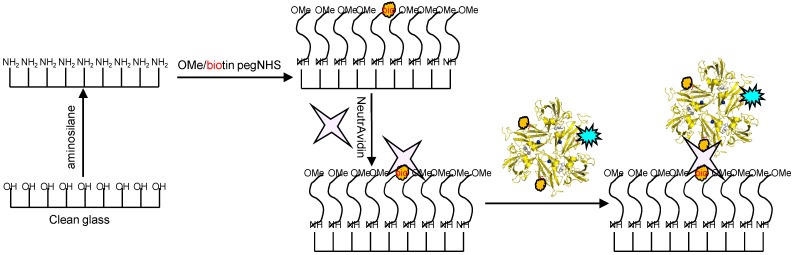
Scheme depicting the functionalization of glass coverslips with aminosilane and PEG linkers and immobilization of SLAC conjugates using the NeutrAvidin-biotin interaction. Reprinted with permission from [43]. Copyright 2014 The American Chemical Society.

**Figure 10:**
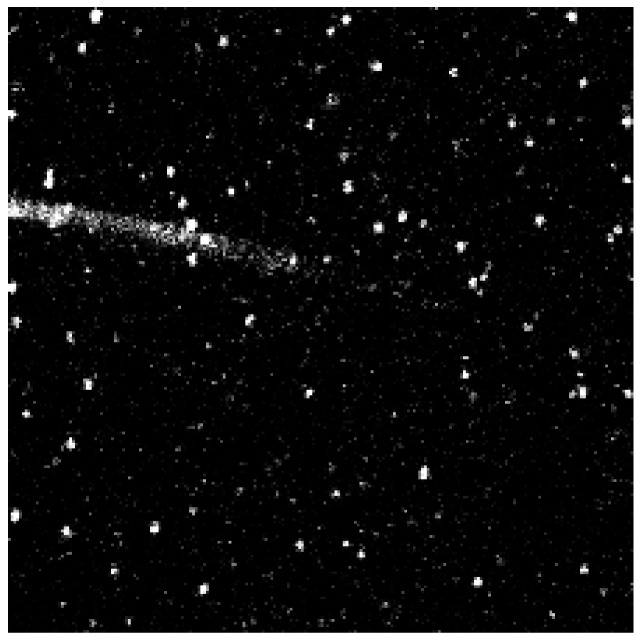
Image (40 × 40 (µm)^2^) taken under a confocal fluorescence microscope of the sample prepared according to the procedure depicted in Figure 9. The bright spots are the individual SLAC molecules. Reprinted with permission from [43]. Copyright 2014 The American Chemical Society.

Glass coverslips of 0.17 mm thickness were chemically modified in the manner depicted in Figure 9. Briefly, the coverslips were silanized with 3-(2-aminoethyl)aminopropyl trimethoxysilane to create a amine terminated surface. This surface was further modified by using PEG linkers which creates an antifouling coating of PEG “brushes” preventing non-specific adsorption of proteins. The PEG linkers were doped with small amounts of biotin-terminated moieties which were utilized to link to the previously prepared SLAC conjugates by incubation with avidin. An image of a sample thus prepared using a confocal microscope is shown in Figure 10.

**Figure 11:**
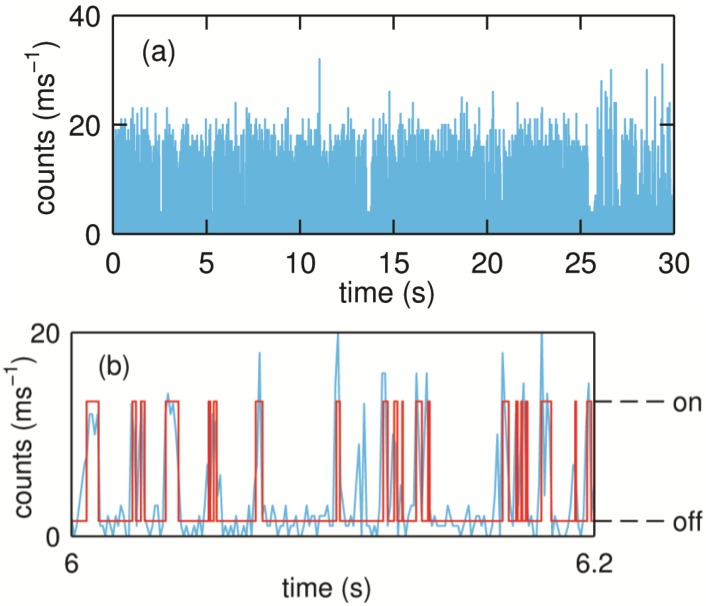
(**a**) Typical binned time trace (1 ms bin time) of a turning over single SLAC molecule. The molecule shows fluctuations between the high and low emission rates as the redox state of T1 Cu changes which can be seen more clearly from a small portion of the trace as shown in (**b**). Reprinted with permission from [43]. Copyright 2014 The American Chemical Society.

When the laser is focused on one of the spots, fluctuations in count rates can be observed depending on the experimental conditions. In the absence of substrate the molecules largely show a steady fluorescence count rate. However, in the presence of substrate, discrete fluctuations in the count rate can be observed as shown in Figure 11. As explained in the section on NiR, these fluctuations can be attributed to the redox switching of the T1 Cu during the turning over of the single SLAC molecules.

**Scheme 3:**
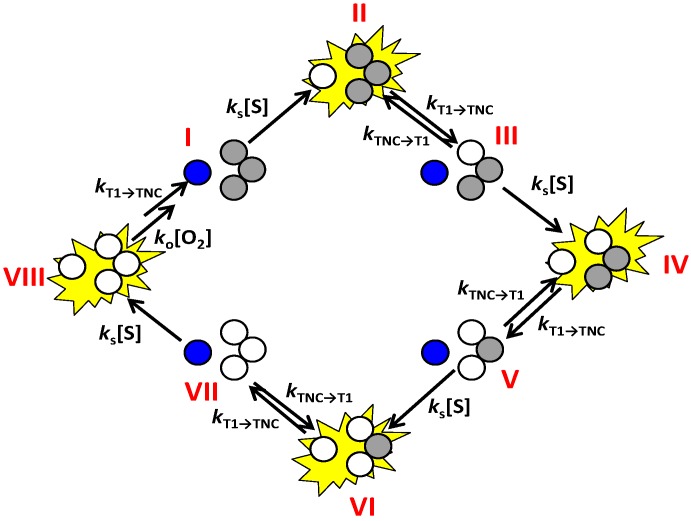
Cartoon representation of the enzyme cycle highlighting the sequential electron transfer steps. Oxidized T1 Cu is depicted in dark blue and the oxidized Cu’s in the TNC in grey. Reduced Cu sites are depicted colorless. The fluorescence emission state of the molecule is shown as a bright yellow star, *i.e*., when the T1 Cu is reduced. Oxygen binding is supposed to occur, in this scheme, in state VIII but may also occur earlier, for instance in state V, VI or VII.

A scheme of the enzyme mechanism highlighting the various ET steps is shown in Scheme 3. From this scheme it can be inferred that the transition from the bright or “on” state to the dim or “off” state is possible only via ET from T1 to TNC.

**Figure 12:**
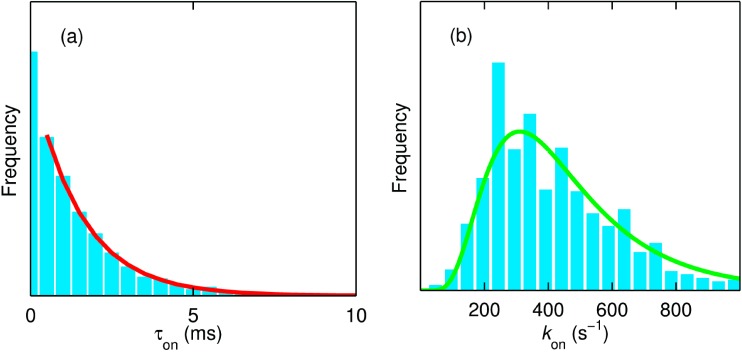
(**a**) The dwell time (τ_on_) distribution of the molecule in the on state from the trace shown in Figure 11a. The number of “on” intervals present in this trace amounts to 2767. The red line is the monoexponential fit to the normalized data with a decay constant (*k*_on_) of 660 s^−1^; (**b**) Distribution of *k*_on_ obtained from ~720 molecules of SLAC. The green line is the fit corresponding to a lognormal distribution with a mean value of 450 s^−1^. The measurements reported in Figure 11 and Figure 12a were made in 20 mM MOPS buffer (pH 7.4) and at 20 °C with DMPD and ascorbate concentrations of 5 mM and 10 mM, respectively. The data reported in panel (**b**) represent measurements that were performed at concentrations of DMPD varying from 0.02 to 5 mM. Reprinted with permission from [43]. Copyright 2014 The American Chemical Society.

A dwell time analysis of single molecule trajectories using the same change point algorithm as used for the analysis of the NiR data (*vide supra*) [34] affords the evaluation of the rate of ET from T1 to TNC (*k*_on_). For the time trajectory shown in Figure 11 this rate amounts to 660 s^−1^ (Figure 12a). It is worth noting that there are at least four forward ET steps (T1→TNC) before completing a single turnover. However, within the time resolution of our experiments and analysis, we observe only a single exponential decay (Figure 12a). Within the time resolution of the experiment all rate constants for the ET from T1→TNC (*k*_T1→TNC_) appear to be similar in magnitude. This would be compatible with relatively minor variations in driving force for ET as a function of the redox state of the catalytic center [44].

We repeated this procedure for ~720 molecules of SLAC where the reducing substrate (*N*,*N*-dimethyl-*p*-phenylenediamine, DMP) concentration was varied between 0.02 and 5 mM. Interestingly, the distribution of these rate constants across many molecules follows a log-normal distribution as one would expect for a normal distribution of the free energy of activation (Figure 12b) [45]. The mean and the spread of the activation energies amounted to 0.347 eV and 0.028 eV, respectively.

It is gratifying to note that the catalytic rate is very different for high (5 mM DMPD) or low (50 μM DMPD) substrate concentrations but that the *k*_on_ distribution is independent of substrate concentration (not shown). This confirms that we are dealing with an intramolecular process, *i.e.*, ET from T1 to TNC.

The off-times were analysed in a similar fashion. According to Scheme 3, *k*_off_ is the sum of two rates: ET from TNC to T1 and a substrate concentration dependent second order rate constant (*k*_s_). At low substrate concentrations (≤50 μM, for example) *k*_off_ approaches the rate of back ET from TNC to T1. It should be kept in mind that the mechanism presented in Scheme 3 is possibly a simplification. The ET steps might be part of a more complex mechanism and may include other intermediates not shown in this scheme. O_2_ binding and reduction, for example, can already take place when the enzyme is reduced by two electrons and can also involve redox processes associated with neighbouring amino acid residues like Y108 [35,36].

**Figure 13:**
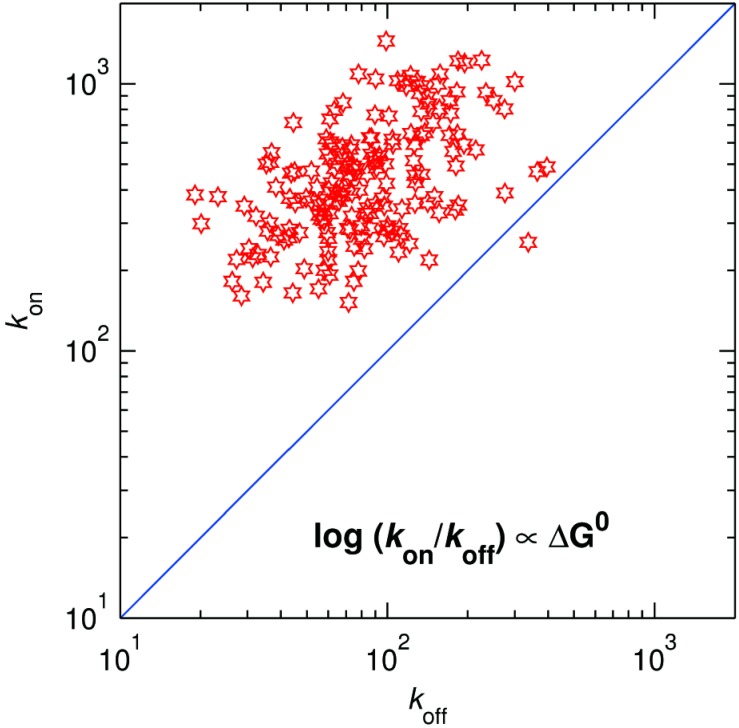
Distribution of *k*_on_ *vs*. *k*_off_ for ~720 individual SLAC molecules. The blue line along the diagonal corresponds with zero driving force.

Thus, the distribution of *k*_off_ at 50 μM DMPD provides the mean and spread of the activation energy associated with the back ET. By taking the ratio of *k*_on_ and *k*_off_ for each molecule, we can now, for the first time, obtain the thermodynamic driving force associated with each molecule. A two-dimensional plot showing the distribution of *k*_on_ and *k*_off_ is presented in Figure 13. As expected, the driving force is generally positive (almost all points lie above the line of zero driving force, *i.e.*, above the diagonal *k*_on_ = *k*_off_). The projection of the points on the antidiagonal in Figure 13 provides the distribution of the driving force. The histogram (not shown) shows that the mean and spread of the driving force amounts to 29 meV and ± 20 meV, respectively.

## 4. Conclusions

The experiments presented above demonstrate that the redox kinetics of an oxido-reductase can be conveniently studied by labelling it with a fluorescent probe. The requirements are that the optical spectrum of the protein varies with its redox state and that the emission spectrum of the fluorophore overlaps with the absorbance of the protein. Once these conditions are fulfilled, it is possible to study enzyme kinetics at the single molecule level. Also, interactions between partners in a redox reaction may be studied: labelling the partners with different dyes allows for studying the correlation of redox events in the two partners. Finally, the (redox) communication between different redox centres in the same protein can be studied in detail. The method is easy to implement and provides, in a relatively simple manner, information that will be difficult to obtain otherwise. Bleaching, blinking and possible redox activity of the dye are known drawbacks that have been documented amply in the literature and strategies have been proposed and developed on how to avoid or mitigate them.

The single molecule findings detailed in the previous pages show that the rates of internal electron transfer exhibit a fairly broad distribution that-in the case of NiR-has been ascribed tentatively to small local disorders in the protein structure and water networks [28]. It is remarkable that on the time scale of the experiment no averaging out of the local structural variations seems to occur. The same can be said of the variations in internal ET rate constants of SLAC. Further, two populations of NiR molecules were identified which were tentatively connected with the two possible reaction pathways of the enzyme. Again, as Figure 6 illustrates, the transition of a molecule from one population to the other appears slow. Finally, the appearance of discrete combinations of fluorescence life time and brightness of a turning-over NiR molecule (Figure 4) points to the occurrence of temporarily stabilized forms of the enzyme. These observations require further attention.

A point worthy of note is that in the example of SLAC it was shown that the molecular distribution of individual driving force and reorganisation energies for ET between the T1 centre and the TNC could be determined. As Figure 13 shows there appears little correlation between driving force and reorganization energy. Future research will amplify on this observation.
